# Evaluation of Safety and Dosimetry of ^177^Lu-DOTA-ZOL for Therapy of Bone Metastases

**DOI:** 10.2967/jnumed.120.255851

**Published:** 2021-08-01

**Authors:** René Fernández, Elisabeth Eppard, Wencke Lehnert, Luis David Jiménez-Franco, Cristian Soza-Ried, Matías Ceballos, Jessica Ribbeck, Andreas Kluge, Frank Rösch, Marian Meckel, Konstantin Zhernosekov, Vasko Kramer, Horacio Amaral

**Affiliations:** 1 Center for Nuclear Medicine and PET/CT, PositronMed, Santiago, Chile;; 2 Positronpharma SA, Santiago, Chile;; 3 ABX-CRO, Dresden, Germany;; 4 Department of Nuclear Medicine, University Medical Center, Hamburg, Germany;; 5 Institute of Nuclear Chemistry, Johannes Gutenberg-University, Mainz, Germany; and; 6 Isotope Technologies Garching GmbH, Munich, Germany

**Keywords:** bone metastasis, bisphosphonate, ^177^Lu-DOTA-ZOL, radionuclide therapy, dosimetry

## Abstract

Palliative treatment of bone metastasis using radiolabeled bisphosphonates is a well-known concept proven to be safe and effective. A new therapeutic radiopharmaceutical for bone metastasis is ^177^Lu-DOTA-zoledronic acid (^177^Lu-DOTA-ZOL). In this study, the safety and dosimetry of a single therapeutic dose of ^177^Lu-DOTA-ZOL were evaluated on the basis of a series of SPECT/CT images and blood samples. **Methods:** Nine patients with exclusive bone metastases from metastatic castration-resistant prostate cancer (mCRPC) (70.8 ± 8.4 y) and progression under conventional therapies participated in this prospective study. After receiving 5,780 ± 329 MBq ^177^Lu-DOTA-ZOL, patients underwent 3-dimensional whole-body SPECT/CT imaging and venous blood sampling over 7 d. Dosimetric evaluation was performed for main organs and tumor lesions. Safety was assessed by blood biomarkers. **Results:** ^177^Lu-DOTA-ZOL showed fast uptake and high retention in bone lesions and fast clearance from the bloodstream in all patients. The average retention in tumor lesions was 0.02% injected activity per gram at 6 h after injection and approximately 0.01% at 170 h after injection. In this cohort, the average absorbed doses in bone tumor lesions, kidneys, red bone marrow, and bone surfaces were 4.21, 0.17, 0.36, and 1.19 Gy/GBq, respectively. The red marrow was found to be the dose-limiting organ for all patients. A median maximum tolerated injected activity of 6.0 GBq may exceed the defined threshold of 2 Gy for the red bone marrow in individual patients (4/8). **Conclusion:** ^177^Lu-DOTA-ZOL is safe and has a favorable therapeutic index compared with other radiopharmaceuticals used in the treatment of osteoblastic bone metastases. Personalized dosimetry, however, should be considered to avoid severe hematotoxicity for individual patients.

## Visual Abstract

**Figure:**
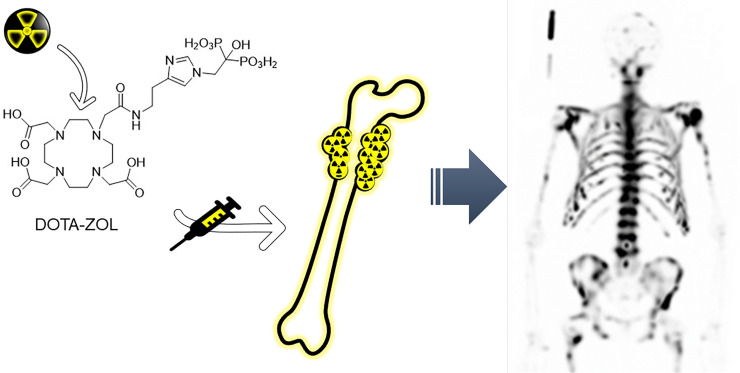

The development of bone metastasis is a well-known complication of prostate cancer in advanced stages. The skeletal metastases often cause severe symptoms reducing the quality of life of the patients significantly (*1*). Currently incurable, skeletal metastases considerably contribute to an increased morbidity and mortality (*2*). Nuclear medicine techniques play a key role in the diagnosis, staging, and treatment of the skeletal metastatic disease.

Compared with most other therapeutic β-emitters, ^177^Lu has favorable physical properties (half-life, 6.7 d; maximum β-energy, 497 keV; γ-energy, 113 keV [6.4%]; 208 keV [11%]) and proven clinical therapeutic utility (*3*,*4*). Moreover, ^177^Lu is commercially available in high specific activities and radionuclide purities. All in all, ^177^Lu is suitable for the treatment of small- and medium-sized tumors and allows for dosimetry and individual treatment planning using scintigraphic and SPECT imaging.

Bisphosphonates are a well-known group of drugs used for treatment of bone disease. Several studies investigated ^177^Lu-labeled bisphosphonates for example, ^177^Lu-EDTMP (*5*) or ^177^Lu-BPAMD (*6*). Although ^177^Lu-EDTMP proved to have high potential for pain palliation (*7*–*9*) as well as favorable radiation dose characteristics compared with other bone-targeting drugs (*10*), its ^68^Ga-analog, with lower accumulation in bone, is not suitable as a diagnostic pair (*11*). In contrast, the DOTA-conjugate BPAMD showed favorable results when labeled with ^177^Lu and ^68^Ga, enabling individualized patient treatment (*12*).

Zoledronic acid, a last-generation bisphosphonate, has shown very high hydroxyapatite affinity and inhibition of the farnesyl diphosphate synthase (*13*). These properties render it an ideal candidate for theranostics, leading to the development of DOTA-zoledronic acid (DOTA-ZOL). Preclinical and first clinical evaluations revealed its high potential (*6*,*10*,*14*,*15*). Biodistribution and skeletal uptake were found to be comparable for the ^68^Ga- or ^177^Lu-labeled compounds (*10*,*16*,*17*). Thus, ^68^Ga-/^177^Lu-DOTA-ZOL (or even ^225^Ac-DOTA-ZOL) provide a set of potential theranostic radiopharmaceuticals, enabling patient-individual dosimetry and pre- and posttherapeutic evaluation.

In this prospective study, the dosimetry and safety of a single therapeutic dose of ^177^Lu-DOTA-ZOL were evaluated in patients with metastatic castration-resistant prostate cancer based on a series of SPECT/CT images and blood samples.

## MATERIALS AND METHODS

### Study Design and Patients

Study approval was obtained from the regional ethics committee board (CEC-SSM-Oriente, permit 20170829). All patients gave written informed consent, and all reported investigations were conducted in accordance with the Helsinki Declaration and with local regulations.

Nine male patients (mean age ± SD, 70.8 ± 8.4 y; range, 57–82 y) were enrolled for ^177^Lu-DOTA-ZOL therapy. Patients had received surgery, radiotherapy, first- or second-line androgen deprivation therapy, or chemotherapy as previous treatments and were on palliative treatment with no other treatment options available at the time of inclusion. The presence of bone metastasis and absence of visceral metastasis was verified by ^68^Ga-PSMA-11 (*n* = 2) or ^18^F-PSMA-1007 (*n* = 7) PET/CT scans within 1 week before therapy. Blood biomarkers (Supplemental Table 1) were evaluated at the day of treatment (baseline), week 4, and week 10 after injection (follow-up). ^177^Lu-DOTA-ZOL was prepared as previously described (*15*). As an intravenous bolus, 5,780 ± 329 MBq (range, 5,215–6,380 MBq) of ^177^Lu-DOTA-ZOL were administered over 6–10 s followed by a saline flush. One of 9 patients (subject 5) needed to be retrospectively excluded from the dosimetric evaluation because of corruption of the SPECT data. A detailed description of radiosynthesis and patient data is given in Supplemental Table 1 (supplemental materials are available at http://jnm.snmjournals.org).

### SPECT/CT Imaging and Blood Sampling

A series of 3-dimensional SPECT/CT imaging was performed to evaluate organ and tumor dosimetry. For each patient, whole-body SPECT/CT scans were acquired (3 bed positions from the top of the head to the upper thighs; 90 projections and 25 s per projection) on a Symbia T2 scanner (Siemens Healthineers) at 1.5 ± 0.5, 6 ± 1, 24 ± 3, 48 ± 3 h, and at 7 ± 1 d after injection. The scanner was equipped with a medium-energy low-penetration collimator. Three energy windows were acquired and used for further processing: a peak window of 20% width centered around the 208 keV energy peak and 2 adjacent corresponding lower and upper scatter energy windows of 10% width each. Quantitative reconstruction of the stitched SPECT images was performed using a 3-dimensional ordered-subset expectation maximization algorithm with 8 iterations and 9 subsets applying uniformity correction, CT-based attenuation correction, energy-based scatter correction, and collimator-detector response modeling.

To yield quantitative images (Bq/mL) a calibration factor was determined from a phantom experiment using an International Electrotechnical Commission National Electrical Manufacturers Association body phantom filled with 765 MBq of ^177^Lu and applied to each patient SPECT dataset.

In addition, venous blood samples of 4 mL were taken at 5 ± 2, 15 ± 5, 30 ± 5 min, 1.5 ± 0.5, 6 ± 1, 24 ± 3, 48 ± 3 h, and 7 ± 1 d after injection, and their activity concentrations were measured to estimate the radiation dose in the red bone marrow.

### Dosimetric Analysis

#### Software

Dosimetric calculations were performed using the QDOSE dosimetry software suite (ABX-CRO) and OLINDA/EXM software, version 1.1 (*18*). Dosimetric calculations for the bone tumor lesions were performed using the spheric model in IDAC-Dose, version 2.1, which accounts for different tissue types, including cortical bone (*19*).

#### Image Processing

All SPECT scans and the corresponding low-dose CT images were analyzed with the QDOSE software. The SPECT images were calibrated by applying the calibration factor determined during camera setup. Coregistration between images was verified and manually corrected when necessary.

#### Source Organs

For dosimetric calculations, the following source organs were included: kidneys, red marrow, cortical bone mineral surface, trabecular bone mineral surface, urinary bladder content, and remainder of body. Red marrow activity uptake was estimated from venous blood sampling.

#### Tumor Definition

Diagnostic PET/CT images were used to select tumor lesions of interest and to determine the lesion volume, using a threshold of 40% of the SUV_max_ in the PET images (*20*).

#### Retrieval of Activity Values and Time–Activity Curves

At each time point, activity values were retrieved from the SPECT images using a threshold-based segmentation algorithm for the kidneys (left and right), urinary bladder content, skeleton (excluding tumor regions), and total body. Manual adjustment of the volumes of interest was applied when necessary. The femora were manually excluded from the volumes of interest for the skeleton. Because the segmented volumes of interest for the total body and the skeleton did not include the legs, the obtained activity values for these organs were scaled by a factor of 1.506 (1.506 = 1/0.664), representing the legs with 33.6% of the total bone mass (*21*).

Mean activity concentration values and lesion volumes were used to determine the tumor activity values as detailed in the supplemental materials.

The time–activity curves for source organs and tumor lesions were determined by the activity values and acquisition times of the SPECT scans.

Time–activity curves for the red marrow were estimated from venous blood sampling as follows (*22*):

$${\text{A}}_{\text{red marrow}}(\text{MBq})=\frac{{\text{AC}}_{\text{blood}}\;(\text{MBq/mL})\;\times \text{RMBLR}\times 1,500\text{g}}{1.05\frac{\text{g}}{\text{mL}}},$$

where A is activity, AC is activity concentration, and RMBLR is red marrow-to-blood activity concentration ratio.

Standard values for the red marrow mass (1,500 g) and density (1.05 g/mL) were considered for this estimation. A RMBLR of 1.0 was used as suggested for ^177^Lu-therapy (*23*).

#### Activity Integration and Safety Dosimetry

Organ and tumor lesion time–activity curves were fitted to a sum of exponential functions, which were integrated from time 0 to infinity to obtain cumulated activity values. Normalized cumulated activity values were calculated by dividing the cumulated activity by the injected activity. Organ normalized cumulated activity values obtained from QDOSE were used for absorbed and effective dose calculations with OLINDA/EXM, version 1.1 (*18*). Additionally, the dose calculator IDAC-Dose 2.1 (*19*) integrated in QDOSE was used for bone surface dose calculations.

The total kidney mass (considering both kidneys) was individually adapted for dose calculations. Kidney volumes were determined on low-dose CT images, and a kidney mass density of 1.06 g/mL was assumed.

Because DOTA-ZOL is a bisphosphonate that accumulates in the bone mineral surface (*24*), the skeleton cumulated activity was distributed between the cortical bone mineral surface (80%) and the trabecular bone mineral surface (20%) (*21*). Bone surface dose was calculated with IDAC-Dose 2.1 as a representative for the dose to the whole skeleton. A total bone mass for the skeleton of 5,500 g was assumed (*21*). Tumor masses were also individually considered and assumed to have the same mass density as cortical bone (1.92 g/mL) (*19*).

Maximum tolerated adsorbed doses of 2 Gy (red marrow), 23 Gy (kidneys), and 10 Gy (bone surfaces) were used to determine the dose-limiting organ (*25*–*27*).

### Evaluation of Side Effects and Toxicity

General safety and adverse effects were assessed at 4 and 10 weeks after injection by blood biomarkers and according to the Common Terminology Criteria for Adverse Events, version 5.0 (*28*). A detailed list of biomarkers for inflammation and kidney and liver function is given in the supplemental materials. Possible hematotoxicity was evaluated by hemoglobin, hematocrit, leukocytes, and platelets, considering grades 3 and 4 as severe.

### Statistical Analysis

All clinical data between baseline and 10 weeks after injection were compared using the paired Wilcoxon test. Two-sided *P* values of less than 0.05 were considered statistically significant. All analyses were performed using Stata software, version 14.

## RESULTS

Nine male patients (70.8 ± 8.4 y) with metastatic castration-resistant prostate cancer with exclusive bone metastases were enrolled for evaluation of safety and dosimetry of a therapeutic dose of 5,780 ± 329 MBq of ^177^Lu-DOTA-ZOL. One patient was retrospectively excluded from the dosimetry analysis because of strong motion artifacts in the SPECT data.

### Biodistribution

**FIGURE 1.**
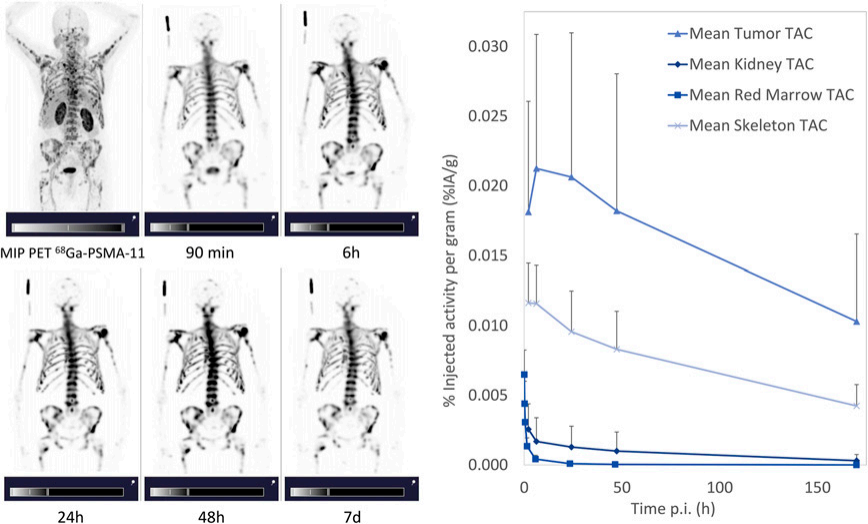
(A) Maximum-intensity projections of PET/CT scan and of whole-body SPECT images at 90 min, 6 h, 24 h, 48 h, and 7 d after injection for representative patient (patient 6). (B) Time–activity curves (TACs) for red marrow, kidneys, skeleton, and tumor lesions.

Representative maximum-intensity projections of whole-body SPECT images, as well as the mean time–activity curves, are presented in Figure 1. The time–activity curves were expressed as percentage injected activity per gram (%IA/g) and not corrected for physical decay of the radionuclide.

**FIGURE 2.**
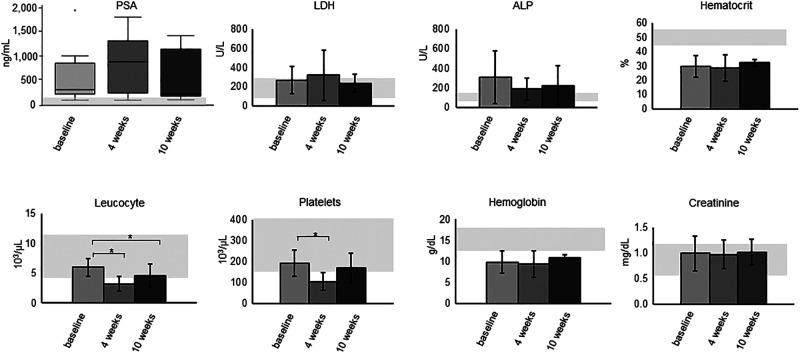
Selected biomarkers at baseline, 4 weeks, and 10 weeks after injection. Gray areas represent normal values. *P < 0.05; point at baseline PSA represents outlier (patient 4). ALP = alkaline phosphatase; LDH = lactate dehydrogenase; PSA = prostate-specific antigen.

**FIGURE 3.**
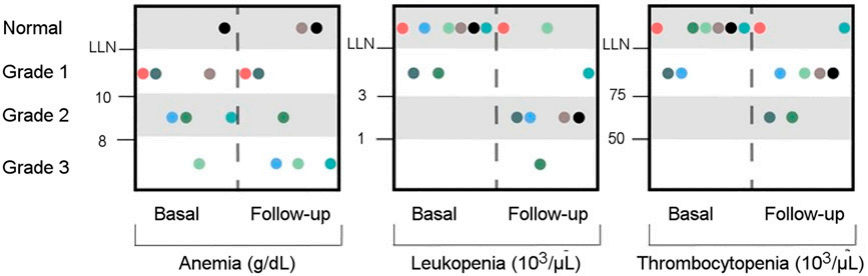
Survey of adverse events according to Common Terminology Criteria for Adverse Events, version 5.0 (28), for 8 of 9 patients before (basal) and 10 weeks after (follow-up) treatment.

Red marrow time–activity curves revealed a fast clearance with low variation within the patient group, with a mean %IA/g of approximately 1.4 × 10^-3^ %IA/g at 1.5 h after injection and 9.7 × 10^−5^ %IA/g at 24 h after injection. In contrast, fast uptake and high retention of ^177^Lu-DOTA-ZOL was observed in the skeleton, with a peak uptake of 9.6 × 10^−3^ ± 2.4 × 10^−3^ %IA/g at 2 h after injection. Even at 170 h after injection, the activity in the skeleton was approximately 3.5 × 10^−3^ ± 1.3 × 10^−3^ %IA/g. The individual skeleton time–activity curves presented a similar shape, with a single exception (patient 9) showing a marked increase between 1.5 and 6 h after injection, The kidney time–activity curves showed a large variation within the patients, and almost no uptake was observed for most of them (6/8).

High retention of ^177^Lu-DOTA-ZOL in the tumor lesions was found, with a mean %IA/g of 2.1 × 10^−2^ at 6 h after injection and approximately 1.0 × 10^-2^ %IA/g at 170 h after injection. However, the shape of the time–activity curves varied not only within the group but also within the same patient, depending on lesion characteristics. Although the highest activity accumulation was found at 6 h after injection for most lesions, for some of them it was either at 1.5 h after injection or at 24 h after injection. As a result, the mean SD for each time point was approximately 50%.

Individual time–activity curves are presented in Supplemental Figures 1–5.

### Safety Dosimetry

Table 1 summarizes the normalized absorbed doses for the organs at risk, namely red marrow, kidneys, and skeleton (bone surfaces), and the normalized whole-body effective dose.

**TABLE 1:** Normalized Absorbed Doses for Organs at Risk (Gy/GBq) and Normalized Effective Dose (mSv/MBq)

Organ	Patient no.								Median	Mean	SD
	1	2	3	4	6	7	8	9			
Red marrow	0.326	0.346	0.564	0.499	0.301	0.206	0.226	0.375	0.336	0.355	0.116
Kidneys	0.063	0.097	0.054	0.053	0.078	0.094	0.250	0.691	0.086	0.172	0.205
Bone surfaces	1.080	1.180	1.980	1.780	1.040	0.635	0.641	1.220	1.130	1.195	0.450
Effective dose	0.143	0.130	0.216	0.177	0.109	0.095	0.106	0.158	0.137	0.142	0.038
Maximum tolerated injected activity (GBq)	6.1	5.8	3.5	4.0	6.6	9.7	8.8	5.3	6.0	6.3	2.0

Dose-limiting organ is red marrow.

The normalized absorbed doses ranged from 0.206 to 0.564 Gy/GBq for the red marrow, from 0.053 to 0.691 Gy/GBq for the kidneys, and from 0.635 to 1.980 Gy/GBq for bone surfaces. The normalized effective doses ranged from 0.095 to 0.216 mSv/MBq. The kidneys showed a much lower normalized absorbed dose than did the red marrow and the bone surfaces, except for 2 patients, patients 8 and 9, who presented an elevated kidney uptake.

Assuming a maximum tolerated dose of 2, 23, and 10 Gy for the red marrow, kidneys, and bone surfaces, respectively, the red marrow was the dose-limiting organ for all patients. The maximum safely injectable activity (i.e., activity leading to a dose that did not surpass any of the defined maximum tolerated doses) ranged from 3.5 to 9.7 GBq, with a median of 6.0 GBq.

### Tumor Dosimetry

The tumor masses determined from the segmented lesion volumes and an assumed density of 1.92 g/mL are presented in detail in Supplemental Table 2. The normalized absorbed doses for the tumor lesions are displayed in Table 2.

**TABLE 2:** Normalized Tumor Doses (Gy/GBq)

Lesion	Patient no.							
	1	2	3	4	6	7	8	9
1	5.02	5.15	3.63	2.85	2.03	8.05	6.13	7.94
2	2.37	3.70	3.00	3.86	2.52	8.94	3.44	11.26^*^
3	5.37	1.72	1.65	2.25	2.79	3.69	1.17	9.27
4	6.98	0.92	1.66	5.91	3.54	6.57	3.26	3.51
5	2.53	1.56	4.10	4.39	4.36	4.26	2.84	NA
Median	5.02	1.72	3.00	3.86	2.79	6.57	3.26	8.60
Mean	4.45	2.61	2.81	3.85	3.05	6.30	3.37	7.99
SD	1.77	1.57	1.00	1.27	0.82	2.05	1.60	2.85

* Dose calculated with SPECT activity threshold of 82% SUV_max_ and volume determined with threshold of 50% SUV_max_ showed very large difference from dose calculated with volume determined in pretherapeutic PET image.

For patient 9, only 4 lesions were defined. Overall tumor statistics were a median of 3.63, a mean of 4.21, and an SD of 2.40.

The normalized absorbed doses for the tumor lesions ranged from 0.92 to 11.26 Gy/GBq. The mean absorbed tumor dose per patient ranged from 2.61 to 7.99 Gy/GBq. The overall variability (∼55%) is acceptable because of the diversity of the lesions (different patients, locations, and sizes).

Therapeutic indices were calculated for the red marrow and bone surfaces as the ratio between the mean absorbed dose for the tumor lesions of each patient and the absorbed doses for these organs. Because of the observed low doses, the kidneys were not considered in these calculations. A detailed table with the therapeutic index values is provided (Supplemental Table 3). Therapeutic indices ranged from 5.0 to 30.6 when considering the red marrow and from 1.4 to 9.9 when considering the bone surfaces.

Furthermore, dosimetric calculations not considering the skeleton (cortical bone mineral surface and trabecular bone mineral surface) as a source organ were performed for comparison. These results revealed that approximately 70% of the red marrow dose was produced by cross-irradiation from the accumulated activity in the skeleton.

### Evaluation of Safety and Adverse Events

The treatment had no statistically significant effect on lactate dehydrogenase, alkaline phosphatase, creatinine, hemoglobin, or hematocrit levels at any follow-up visit. A significant reduction was observed for leukocytes after 4 and 10 weeks after injection and for platelets after 4 weeks after injection (*P* < 0.05). Nevertheless, the initial effect on leukocytes and platelets was transitory, and patients showed recovery from weeks 4 to 10. We observed grade 3 anemia in 3 of 9 patients (1 already had grade 3 anemia previously; 2 had grade 2) and grade 3 leukopenia in 1 of 9 patients (from grade 1 previously). Further, no patient experienced relevant xerostomia, fatigue, nausea, loss of appetite, nephrotoxicity, or hepatotoxicity.

## DISCUSSION

Dosimetry and safety evaluations of ^177^Lu-DOTA-ZOL were performed for 9 patients with metastatic castration-resistant prostate cancer.

For all patients, the red marrow was the dose-limiting organ, allowing maximum injected activities of 3.5–9.7 GBq. Overall, a median injected activity of 6 GBq was calculated as the maximum activity tolerated without exceeding the defined threshold of 2 Gy for the red marrow. However, administration of 6 GBq of ^177^Lu-DOTA-ZOL may lead to red marrow doses of 3 Gy or higher in some patients. Because absorbed doses higher than 3 Gy have been associated with more severe side effects (*29*), a more conservative treatment administering only 3.5 GBq may guarantee that a red marrow dose of 2 Gy is not exceeded in any of the patients. Here, a conservative RMBLR value of 1.0 was applied as suggested for ^177^Lu-based peptide receptor radionuclide therapy (*30*). The contribution of red marrow activity to the normalized dose is approximately 30%; therefore, a RMBLR value of 0.36, as used in other studies (*31*), would have resulted in an approximately 19% lower absorbed doses. In addition, the presented results are based on calculations using the widely accepted OLINDA/EXM 1.1, which makes specific assumptions for red marrow dose calculations (*30*). Results calculated with IDAC-Dose 2.1 and IDAC-Dose 1.0 would have been 12% and 65% higher, respectively. Thus, the use of different dose calculators or different assumptions can potentially lead to different results. Furthermore, tumor uptake was excluded from general bone uptake, but depending on their location, bone lesions may also contribute to the red marrow dose, which could have been underestimated in this study. When including the tumor activity as part of the bone activity, the red marrow dose was 8% to 33% higher (median, 19% higher). Certainly, to achieve optimal results and to avoid severe side effects, treatment with ^177^Lu-DOTA-ZOL should be carefully planned and monitored in a personalized approach in terms of injected activity and number of cycles.

In contrast, the kidneys, because of their low uptake and fast clearance, do not pose a limitation. With a kidney dose threshold of 23 Gy, the maximum tolerated injected activities ranged from 33.3 to 431.5 GBq.

The tolerable dose limit for the skeleton, 10 Gy, may have a high uncertainty because it was determined in a retrospective study without dosimetry (*25*) and after radiation exposure from a combination of ^226^Ra (α-emitter) and ^228^Ra (β^−^-emitter) (*26*). Additionally, other authors have suggested different dose thresholds for certain parts of the skeleton (*32*).

A previous safety dosimetry evaluation for ^177^Lu-DOTA-ZOL by Khawar et al. (*15*) reported absorbed doses to the red marrow, kidneys, and osteogenic cells that were 30% higher (mean, 0.461 vs. 0.355 mGy/MBq), 185% higher (mean, 0.490 vs. 0.172 mGy/MBq), and 35% lower (mean, 3.30 vs. 5.14 mGy/MBq), respectively, than the values found in this work. Differences in absorbed doses to red marrow and osteogenic cells can be expected because of different assumptions in bone activity calculation. More importantly, the current study used 3-dimensional SPECT imaging whereas the dosimetry evaluation by Khawar et al. was based on planar imaging (*15*). In particular, for bone-seeking agents, SPECT-based dosimetry has the advantage that the organs and structures of interest can be accurately segmented without including activity from overlapping structures. This advantage also enabled tumor dosimetry, which had not been performed previously for ^177^Lu-DOTA-ZOL.

In Table 3, ^177^Lu-DOTA-ZOL is directly compared with other therapeutic radiopharmaceuticals for bone palliation.

**TABLE 3:** Comparison of ^177^Lu-DOTA-ZOL to Other Radiopharmaceuticals Used for Bone Metastasis Treatment

Parameter	^177^Lu-DOTA-ZOL	^89^SrCl_2_ (*33*)	^153^Sm-EDTMP (*34*)	^177^Lu-EDTMP (*34*)	^188^Re-HEDP (*35*)	^223^Ra-Cl_2_^*^
Tumor lesion	4.21 ± 2.40	233 ± 166	6.22 ± 4.21	6.92 ± 3.92	3.83 ± 2.01	179.8 (68–490) (*36*)
Red marrow	0.36 ± 0.12	18.9	1.41 ± 0.6	0.83 ± 0.21	0.61 ± 0.21	73.9 (*37*)
Bone surface	1.19	30.2	7.8	NA	1.403	739.1 (*37*)
Tumor–to–red-marrow dose ratio	13.9	12.3	4.40	8.31	6.28	2.4
Tumor–to–bone-surface dose ratio	3.5	7.7	0.8	NA	2.7	0.2

* ^223^Ra is α-emitter; therefore, relative biological effectiveness factor of 5 needs to be applied for comparison with β^−^-emitters (relative biological effectiveness = 1).

NA = not applicable.

Values are normalized absorbed doses (Gy/GBq) as mean ± SD. Tumor–to–red-marrow dose ratio and tumor–to–bone-surface dose ratio were calculated as mean of dose ratios for each patient. Bone surface dose was estimated using bone-to-red marrow dose ratios of 1.6 for ^89^SrCl_2_ (*38*), 5.5 for ^153^Sm-EDTMP (*38*), and 2.3 for ^186^Re-HEDP (*38*). Dose-limiting organ was red marrow.

Although a direct comparison of multiple radiopharmaceuticals is challenging because of the different methodologies, ^177^Lu-DOTA-ZOL may have a more favorable therapeutic index than the other radiopharmaceuticals (Table 3). Furthermore, ^177^Lu-DOTA-ZOL shows the second highest tumor-to-bone surface dose ratio after ^89^SrCl_2_. Accordingly, it can be assumed that ^177^Lu-DOTA-ZOL may lead to fewer bone-related side effects than comparable therapies.

Although the methods applied to define the tumor volumes and activities may lead to additional uncertainties, the obtained tumor doses for ^177^Lu-DOTA-ZOL are in the same range as published data for other β^−^-emitters (except for ^89^SrCl_2_) (Table 3).

## CONCLUSION

Palliative treatment of bone metastasis using radiolabeled bisphosphonates is proven to be safe and effective. ^177^Lu-DOTA-ZOL is a new theranostic radiotracer for this indication with favorable pharmacokinetics. In this study, we evaluated the safety and dosimetry of a single therapeutic dose of ^177^Lu DOTA-ZOL, which showed high uptake and retention in bone lesions. Although ^177^Lu-DOTA-ZOL therapy was well tolerated and had no observable severe adverse events, the injected activities and the number of administered cycles need to be carefully determined for each patient to avoid severe hematotoxicity. The obtained results and the observed favorable therapeutic index, compared with established bone-targeting agents, underline the clinical potential and benefit of ^177^Lu DOTA-ZOL for therapy for patients with metastatic castration-resistant prostate cancer.

## DISCLOSURE

Patent applications on conjugated bisphosphonates for the diagnosis and treatment of bone diseases have been licensed to Isotope Technology Munich (ITM) AG. No other potential conflict of interest relevant to this article was reported.

**KEY POINTS**

**QUESTION:** What is the maximum tolerated injected activity of ^177^Lu-DOTA-ZOL for the dose-limiting organ?
**PERTINENT FINDINGS:** For all patients treated, the red marrow was the dose-limiting organ, with a median injected activity of 6 GBq calculated as the maximum activity tolerated.
**IMPLICATIONS FOR PATIENT CARE:** The results of this study and the favorable therapeutic index as compared with established bone-targeting agents underline the clinical potential of ^177^Lu-DOTA-ZOL and its benefit for treatment of patients with metastatic castration-resistant prostate cancer.

## References

[1] Hensel J Thalmann GN. Biology of bone metastases in prostate cancer. Urology. 2016;92:6–13. 26768714 10.1016/j.urology.2015.12.039

[2] Ulmert D Solnes L Thorek DL. Contemporary approaches for imaging skeletal metastasis. Bone Res. 2015;3:15024. 26273541 10.1038/boneres.2015.24 PMC4502405

[3] Frilling A Weber F Saner F. Treatment with ^90^Y- and ^177^Lu-DOTATOC in patients with metastatic neuroendocrine tumors. Surgery. 2006;140:968–976. 17188146 10.1016/j.surg.2006.07.030

[4] Ahmadzadehfar H Eppard E Kürpig S. Therapeutic response and side effects of repeated radioligand therapy with ^177^Lu-PSMA-DKFZ-617 of castrate-resistant metastatic prostate cancer. Oncotarget. 2016;7:12477–12488. 26871285 10.18632/oncotarget.7245 PMC4914299

[5] Chakraborty S Das T Banerjee S. ^177^Lu-EDTMP: a viable bone pain palliative in skeletal metastasis. Cancer Biother Radiopharm. 2008;23:202–213. 18454689 10.1089/cbr.2007.374

[6] Pfannkuchen N Meckel M Bergmann R. Novel radiolabeled bisphosphonates for PET diagnosis and endoradiotherapy of bone metastases. Pharmaceuticals (Basel). 2017;10:45. 10.3390/ph10020045 PMC5490402 28524118

[7] Agarwal KK Singla S Arora G Bal C. ^177^Lu-EDTMP for palliation of pain from bone metastases in patients with prostate and breast cancer: a phase II study. Eur J Nucl Med Mol Imaging. 2015;42:79–88. 25070686 10.1007/s00259-014-2862-z

[8] Thapa P Nikam D Das T Sonawane G Agarwal JP Basu S. Clinical efficacy and safety comparison of ^177^Lu-EDTMP with ^153^Sm-EDTMP on an equidose basis in patients with painful skeletal metastases. J Nucl Med. 2015;56:1513–1519. 26315829 10.2967/jnumed.115.155762

[9] Yuan J Liu C Liu X. Efficacy and safety of ^177^Lu-EDTMP in bone metastatic pain palliation in breast cancer and hormone refractory prostate cancer: a phase II study. Clin Nucl Med. 2013;38:88–92. 23334120 10.1097/RLU.0b013e318279bf4d

[10] Meckel M Bergmann R Miederer M Roesch F. Bone targeting compounds for radiotherapy and imaging: *Me(III)-DOTA conjugates of bisphosphonic acid, pamidronic acid and zoledronic acid. EJNMMI Radiopharm Chem. 2017;1:14. 10.1186/s41181-016-0017-1 PMC5843815 29564390

[11] Mirzaei A Jalilian AR Badbarin A. Optimized production and quality control of ^68^Ga-EDTMP for small clinical trials. Ann Nucl Med. 2015;29:506–511. 25903357 10.1007/s12149-015-0971-9

[12] Meckel M Nauth A Timpe J. Development of a ^177^LuBPAMD labeling kit and an automated synthesis module for routine bone targeted endoradiotherapy. Cancer Biother Radiopharm. 2015;30:94–99. 25714451 10.1089/cbr.2014.1720

[13] Dalle Carbonare L Zanatta M Gasparetto A Valenti MT. Safety and tolerability of zoledronic acid and other bisphosphonates in osteoporosis management. Drug Healthc Patient Saf. 2010;2:121–137. 21701624 10.2147/DHPS.S6285 PMC3108695

[14] Khawar A Eppard E Roesch F. Preliminary results of biodistribution and dosimetric analysis of [^68^Ga]Ga-DOTAZOL: a new zoledronate-based bisphosphonate for PET/CT diagnosis of bone diseases. Ann Nucl Med. 2019;33:404–413. 30877560 10.1007/s12149-019-01348-7

[15] Khawar A Eppard E Roesch F. Biodistribution and post-therapy dosimetric analysis of [^177^Lu]Lu-DOTAZOL in patients with osteoblastic metastases: first results. EJNMMI Res. 2019;9:102. 31781962 10.1186/s13550-019-0566-x PMC6882969

[16] Chopra A. ^68^Ga-labeled (4-{(bis(phosphonomethyl))carbamoylmethyl}-7,10-bis(carboxymethyl)-1,4,7,10-tetraazacyclododec-1-yl)acetic acid (BPAMD). Molecular Imaging and Contrast Agent Database (MICAD) website. https://www.ncbi.nlm.nih.gov/books/NBK114339/. Created September 18, 2012. Updated November 21, 2012. Accessed July 19, 2021.

[17] Pfannkuchen N Bausbacher N Pektor S Miederer M Rosch F. In vivo evaluation of ^225^AcAc-DOTAZOL for α-therapy of bone metastases. Curr Radiopharm. 2018;11:223–230. 29866026 10.2174/1874471011666180604083911

[18] Stabin MG Sparks RB Crowe E. OLINDA/EXM: the second-generation personal computer software for internal dose assessment in nuclear medicine. J Nucl Med. 2005;46:1023–1027. 15937315

[19] Andersson M Johansson L Eckerman K Mattsson S. IDAC-Dose 2.1, an internal dosimetry program for diagnostic nuclear medicine based on the ICRP adult reference voxel phantoms. EJNMMI Res. 2017;7:88. 29098485 10.1186/s13550-017-0339-3 PMC5668221

[20] Boellaard R O’Doherty MJ Weber WA. FDG PET and PET/CT: EANM procedure guidelines for tumour PET imaging—version 1.0. Eur J Nucl Med Mol Imaging. 2010;37:181–200. 19915839 10.1007/s00259-009-1297-4 PMC2791475

[21] Basic anatomical and physiological data for use in radiological protection: reference values—a report of age- and gender-related differences in the anatomical and physiological characteristics of reference individuals. ICRP publication 89. Ann ICRP. 2002;32:5–265. 14506981

[22] Hindorf C Glatting G Chiesa C Lindén O Flux G. EANM Dosimetry Committee guidelines for bone marrow and whole-body dosimetry. Eur J Nucl Med Mol Imaging. 2010;37:1238–1250. 20411259 10.1007/s00259-010-1422-4

[23] Forrer F Krenning EP Kooij PP. Bone marrow dosimetry in peptide receptor radionuclide therapy with ^177^Lu-DOTA^0^,Tyr^3^octreotate. Eur J Nucl Med Mol Imaging. 2009;36:1138–1146. 19247653 10.1007/s00259-009-1072-6 PMC2691529

[24] Eppard E Meisenheimer M de La Fuente A Kurpig S Essler M Roesch F. Radiolabelling of DOTAMZOL with ^68^Ga and ^44^Sc for clinical application. Endocr Abstr. 2016;47:OC34.

[25] Flux GD. Imaging and dosimetry for radium-223: the potential for personalized treatment. Br J Radiol. 2017;90:20160748. 28654303 10.1259/bjr.20160748 PMC5858794

[26] Rowland RE. Radium in humans: a review of U.S. studies; 1994.

[27] Kabasakal L AbuQbeitah M Aygun A. Pre-therapeutic dosimetry of normal organs and tissues of ^177^Lu-PSMA-617 prostate-specific membrane antigen (PSMA) inhibitor in patients with castration-resistant prostate cancer. Eur J Nucl Med Mol Imaging. 2015;42:1976–1983. 26227531 10.1007/s00259-015-3125-3

[28] Common terminology criteria for adverse events (CTCAE) version 5.0. National Cancer Institute website. https://ctep.cancer.gov/protocolDevelopment/electronic_applications/docs/CTCAE_v5_Quick_Reference_8.5x11.pdf. Published November 27, 2017. Accessed June 21, 2021.

[29] Loke KS Padhy AK Ng DC Goh AS Divgi C. Dosimetric considerations in radioimmunotherapy and systemic radionuclide therapies: a review. World J Nucl Med. 2011;10:122. 22144871 10.4103/1450-1147.89780 PMC3227338

[30] Stabin MG Siegel JA Sparks RB Eckerman KF Breitz HB. Contribution to red marrow absorbed dose from total body activity: a correction to the MIRD method. J Nucl Med. 2001;42:492–498. 11337528

[31] Delker A Fendler WP Kratochwil C. Dosimetry for ^177^Lu-DKFZ-PSMA-617: a new radiopharmaceutical for the treatment of metastatic prostate cancer. Eur J Nucl Med Mol Imaging. 2016;43:42–51. 26318602 10.1007/s00259-015-3174-7

[32] Emami B Lyman J Brown A. Tolerance of normal tissue to therapeutic irradiation. Int J Radiat Oncol Biol Phys. 1991;21:109–122. 2032882 10.1016/0360-3016(91)90171-y

[33] Robinson RG Blake GM Preston DF. Strontium-89: treatment results and kinetics in patients with painful metastatic prostate and breast cancer in bone. Radiographics. 1989;9:271–281. 2467331 10.1148/radiographics.9.2.2467331

[34] Sharma S Singh B Koul A Mittal BR. Comparative therapeutic efficacy of ^153^Sm-EDTMP and ^177^Lu-EDTMP for bone pain palliation in patients with skeletal metastases: patients’ pain score analysis and personalized dosimetry. Front Med (Lausanne). 2017;4:46. 28507988 10.3389/fmed.2017.00046 PMC5410571

[35] Liepe K Hliscs R Kropp J Runge R Knapp FF Franke W-G. Dosimetry of ^188^Re-hydroxyethylidene diphosphonate in human prostate cancer skeletal metastases. J Nucl Med. 2003;44:953–960. 12791825

[36] Pacilio M Ventroni G de Vincentis G. Dosimetry of bone metastases in targeted radionuclide therapy with alpha-emitting ^223^Ra-dichloride. Eur J Nucl Med Mol Imaging. 2016;43:21–33. 26266887 10.1007/s00259-015-3150-2

[37] Lassmann M Eberlein U. Targeted alpha-particle therapy: imaging, dosimetry, and radiation protection. Ann ICRP. 2018;47:187–195. 29664326 10.1177/0146645318756253

[38] Atkins HL Srivastava SC. Radiopharmaceuticals for bone malignancy therapy. J Nucl Med Technol. 1998;26:80–83. 9604827

